# Evaluation of pathogenic variants detected in high homology regions of the *PMS2* gene. How effective is long-range PCR?

**DOI:** 10.3389/fonc.2024.1390221

**Published:** 2024-06-18

**Authors:** Daniele Paixão, Thalitta Hetamaro Ayala Lima, Rafaela Rogério Floriano de Souza, Juliana Emilia Prior Carnavalli, Clarissa Gondim Picanço-Albuquerque, Isabelle Joyce de Lima Silva-Fernandes, Paulo Goberlânio de Barros Silva, Miguel Mitne-Neto, Caroline Mônaco Moreira, Wagner Antônio da Rosa Baratela

**Affiliations:** ^1^ Fleury Medicina e Saúde, Grupo Fleury, São Paulo, Brazil; ^2^ Laboratório de Biologia Molecular, Hospital Haroldo Juaçaba, Instituto do Câncer do Ceará, Fortaleza, Brazil; ^3^ Instituto Paulo Gontijo (IPG), São Paulo, Brazil

**Keywords:** long-range PCR, *PMS2* gene, *PMS2CL* pseudogene, Lynch syndrome, next-generation sequencing

## Abstract

**Introduction:**

Lynch syndrome (LS) is an inherited cancer predisposition syndrome characterized by a high risk of colorectal and extracolonic tumors. Germline pathogenic variants (GPV) in the *PMS2* gene are associated with <15% of all cases. The *PMS2CL* pseudogene presents high homology with *PMS2*, challenging molecular diagnosis by next-generation sequencing (NGS). Due to the high methodological complexity required to distinguish variants between *PMS2* and *PMS2CL*, most laboratories do not clearly report the origin of this molecular finding.

**Objective:**

The aim of this study was to confirm the GPVs detected by NGS in regions of high homology segments of the *PMS2* gene in a Brazilian sample.

**Methods:**

An orthogonal and gold standard long-range PCR (LR-PCR) methodology to separate variants detected in the *PMS2* gene from those detected in the pseudogene.

**Results:**

A total of 74 samples with a *PMS2* GPV detected by NGS in exons with high homology with *PMS2CL* pseudogene were evaluated. The most common was NM_000535.6:c.2182_2184delinsG, which was previously described as deleterious mutation in a study of African-American patients with LS and has been widely reported by laboratories as a pathogenic variant associated with the LS phenotype. Of all GPVs identified, only 6.8% were confirmed by LR-PCR. Conversely, more than 90% of GPV were not confirmed after LR-PCR, and the diagnosis of LS was ruled out by molecular mechanisms associated with *PMS2.*

**Conclusion:**

In conclusion, the use of LR-PCR was demonstrated to be a reliable approach for accurate molecular analysis of *PMS2* variants in segments with high homology with *PMS2CL*. We highlight that our laboratory is a pioneer in routine diagnostic complementation of the *PMS2* gene in Brazil, directly contributing to a more assertive molecular diagnosis and adequate genetic counseling for these patients and their families.

## Introduction

Colorectal cancer (CRC) is the second most frequent cancer among men and women in Brazil, corresponding approximately to more than 45 thousand new cases per year (1). Approximately 5% of CRCs are associated with germline variants, and Lynch syndrome (LS) is the most prevalent cause of hereditary CRC and is an autosomal dominant disorder related to monoallelic germline pathogenic variants (GPVs) in DNA mismatch repair (MMR) genes *MLH1, MSH2, MSH6*, and *PMS2*, and deletions in the *EPCAM* gene (2–4). It is clinically characterized by predisposition to a broad spectrum of tumors, including early-onset CRC and extracolonic tumors, including endometrial, ovarian, gastric, ureter, renal pelvis, pancreatic, prostate, biliary tract, central nervous system, and small bowel (5, 6).

While GPV in *MLH1* and *MSH2* genes account for almost 70% of LS cases, mutations in *PMS2* contribute to <15% (7, 8). Molecular testing of *PMS2* is challenging due to high homology of *PMS2* gene to its counterpart *PMS2CL* pseudogene, which is considered biologically inactive. Both are located on chromosome 7, and interpreting the clinical relevance of variants detected in these regions is essential for patients’ follow-up and is considered an important challenge nowadays (9).

The *PMS2CL* pseudogene presents high homology (>98%) with *PMS2*, with the greatest identity being found over exon 9 and between exons 11 and 15 (10, 11). NGS is able to identify variants along all the coding segments of this gene; however, mapping and variant calling pipelines struggle to differentiate whether a variant is present in the gene or in the pseudogene. Because of the high methodological complexity required to distinguish variants between *PMS2* and *PMS2CL*, most laboratories do not clearly report the origin of this molecular finding.

Thus, due to the extreme importance of correctly reporting reliable variants in the *PMS2* gene, the aim of this study was to confirm the GPVs detected by NGS in regions of high homology segments of the *PMS2* gene in a Brazilian sample using the orthogonal and gold standard long-range PCR (LR-PCR) methodology to separate variants detected in the *PMS2* gene from those detected in the pseudogene. This strategy will prompt reliable results that will directly contribute to appropriate clinical management.

## Methods

### Samples selection

We selected a total of 74 samples with *PMS2* GPV detected by NGS Panels for Hereditary Cancer, performed at Fleury Genomics laboratory between December 2018 and August 2021. Samples were selected regardless of the personal or familial history of cancer. All participants provided informed consent before blood withdrawal or saliva collection. The study protocol was reviewed and approved by the Human Research Ethics Committee of Fleury Group (protocol number NP_614; Plataforma Brasil CAAE# 56961222.6.0000.5474; Fleury# 5.833.008).

### DNA samples and amplification

Genomic DNA was extracted from peripheral blood, saliva, or swab samples using QIASymphony (QIAGEN, Inc.) with the QIASymphony DNA Mini Kit, QIAmp DNA Blood Mini Kit, and QIAamp DNA Blood Mini Kit (all from QIAGEN, Inc.), respectively. DNA fragmentation was followed by indexing, capture with custom probes, and enrichment of the regions of interest. Paired-end NGS was performed using Illumina platforms, either NovaSeq or NextSeq500 (Illumina, Inc., San Diego, CA, USA). Bioinformatics pipelines were used to perform the alignment and detection of variants based on the GRCh37 (Hg19) version of the Human Genome. The data generated by sequencing were analyzed using local customized bioinformatics processes.

All hereditary cancer predisposition panel data evaluated in this study were generated using the NGS approach. Considering the limitations of the current methodology, all the detected *PMS2* GPV variants were confirmed by the orthogonal and gold standard methodology, LR-PCR, followed by nested PCR. The variants detected in the *PMS2* gene were initially detected using the NGS methodology, which presents methodological limitations for evaluating regions that overlap with pseudogenes because the reads have an average size of 150 bp in the sequencing used (Illumina, Inc., San Diego, CA, USA), which is insufficient to distinguish genes from pseudogenes, considering that some of these intervals have more than 95% homology, requiring complementation using orthogonal methodology (LR_PCR).

The LR-PCR technique described by Vaughn et al. (2010) was employed. This technique involves an initial amplification of regions not anchored in regions of high homology with the pseudogene, followed by a new amplification of only the region to be evaluated. For this, we used a set of specific primers aimed at the amplification of *PMS2* gene, in a similar approach as previously published in the literature.

The protocol used for LR-PCR was previously described by Vaughn et al. (2010) with some adaptations (i.e., Herculase II fusion DNA polymerase enzyme was used, from Agilent Technologies, Santa Clara). For amplification of the region under investigation, the primers listed in **Table 1** were initially used for LR-PCR. For this reaction, we used a high-complexity long-range DNA polymerase enzyme (Herculase II fusion DNA polymerase—Agilent Technologies, Santa Clara). Subsequently, nested PCR was performed using the primers described in **Table 2** (12–14).

**Table 1:** Set of primers used for long-range PCR.

Primer—long range	Sequence
PMS2_LR_exons_1–5_F	ACGTCGAAAGCAGCCAATGGGAGTT
PMS2_LR_exons_1–5_R	CTTCCACCTGTGCATACCACAGGCT
PMS2_LR_exons_7–9_F	GGTCCAGGTCTTACATGCATACTGT
PMS2_LR_exons_7–9_R	CTGACTGACATTTAGCTTGTTGACA
PMS2_LR_exons_11–15_F	GCGTTGATATCAATGTTACTCCAGA
PMS2_LR_exons_11–15_R	CCTTCCATCTCCAAAACCAGCAAGA
PMS2_LR_exon 13–15_F	AAAATTAGTCAGACTTGATGGTGTG
PMS2_LR3_exon 11–12_R	AGTAGTCAGGGTAAAACATTCCAGT

LR, long range; F, forward; R, reverse.

Example: PMS2_LR_exons_1–5_F and PMS2_LR_exons_1–5_R: set of primers used for evaluating exons 1–5 in the PMS2 gene, forward (F) and reverse (R) primers.

**Table 2:** Set of primers used for nested PCR.

Primer	Sequence
M13F_PMS2_Exon_1_F	TGTAAAACGACGGCCAGTACGTCGAAAGCAGCCAATGGGAGTT
M13R_PMS2_Exon_1_R	CAGGAAACAGCTATGACCCAGGTAGAAAGGAAATGCATTCAGT
M13F_PMS2_Exon_2_F	TGTAAAACGACGGCCAGTACAGTGTTGAGTCATTTCCCACAGT
M13R_PMS2_Exon_2_R	CAGGAAACAGCTATGACCTTCTTAGCATAACACCTGCCTGGCA
M13F_PMS2_Exons_3_4_F	TGTAAAACGACGGCCAGTCTGGGCTAGTAAATAGCCAGAAAG
M13R_PMS2_Exons_3_4_R	CAGGAAACAGCTATGACCTATGACTTAGATTGGCAGCGAGACA
M13F_PMS2_Exon_5_F	TGTAAAACGACGGCCAGTCTTGATTATCTCAGAGGGATCGTCA
M13R_PMS2_Exon_5_R	CAGGAAACAGCTATGACCTCTCACTGTGTTGCCCAGTCCTAAT
M13F_PMS2_Exon_6_F	TGTAAAACGACGGCCAGTTGCTTCCCTTGATTTGTGCGATGAT
M13R_PMS2_Exon_6_R	CAGGAAACAGCTATGACCCTACTGGAAGGGACAATGGAAACC
M13F_PMS2_Exon_7_F	TGTAAAACGACGGCCAGTATTGTACTCCAGCCTGGGCAATAG
M13R_PMS2_Exon_7_R	CAGGAAACAGCTATGACCATTGTAGTTCTCTTGCCAGCAATC
M13F_PMS2_Exon_8_F	TGTAAAACGACGGCCAGTAGATTTGGAGCACAGATACCCGTGA
M13R_PMS2_Exon_8_R	CAGGAAACAGCTATGACCTGCGGTAGACTTCTGTAAATGCACA
M13F_PMS2_Exon_9_F	TGTAAAACGACGGCCAGTCCTTCTAAGAACATGCTGGTTGGTT
M13R_PMS2_Exon_9_R	CAGGAAACAGCTATGACCATCTCATTCCAGTCATAGCAGAGCT
M13F_PMS2_Exon_10_F	TGTAAAACGACGGCCAGTAATTAGCCAGTGTGGTGGCACTTG
M13R_PMS2_Exon_10_R	CAGGAAACAGCTATGACCAGCTTTAGAAGCTGTTTGTACAC
M13F_PMS2_Exon_11a_F	TGTAAAACGACGGCCAGTTCACATAAGCACGTCCTCTCACCAT
M13R_PMS2_Exon_11a_R	CAGGAAACAGCTATGACCCTGGTTTGAATGGCAGTCCACATC
M13F_PMS2_Exon_11b_F	TGTAAAACGACGGCCAGTTCGCAGGAACATGTGGACTCTCAG
M13R_PMS2_Exon_11b_R	CAGGAAACAGCTATGACCGCAACAGAGCAAGACTCTGTCTCAA
M13F_PMS2_Exon_12_F	TGTAAAACGACGGCCAGTTTACAGTGTTCTATAACATAATCAG
M13R_PMS2_Exon_12_R	CAGGAAACAGCTATGACCAGTAGATACAAGGTCTTGCTGTGTT
M13F_PMS2_Exon_13_F	TGTAAAACGACGGCCAGTGTGACACTTAGCTGAGTAGTGTTGT
M13R_PMS2_Exon_13_R	CAGGAAACAGCTATGACCATGTTAGCCAGGCTGGTCTCAAACT
M13F_PMS2_Exon_14_F	TGTAAAACGACGGCCAGTGGTCTGTATCTCCTGACCTCATGAT
M13R_PMS2_Exon_14_R	CAGGAAACAGCTATGACCGCACGTAGCTCTCTGTGTAAAATGA
M13F_PMS2_Exon_15_F	TGTAAAACGACGGCCAGTGCTGAGATCTAGAACCTAGGCTTCT
M13R_PMS2_Exon_15_R	CAGGAAACAGCTATGACCACACACGAGCGCATGCAAACATAGA

M13, M13 primers (forward and reverse); F, forward; R, reverse.

We used a subset of primers described in **Table 1** as amplification primers in a final volume of 50 μL, containing 150 ng of DNA, 0.5 μM each primer (Thermo Fisher Scientific Inc., Waltham, MA), 1.25 μL Herculase II fusion DNA polymerase, 1× PCR buffer (5× Herculase II reaction buffer), and 400 μM each dNTP (all from Agilent Technologies, Santa Clara). Cycling conditions were as follows: initial denaturation of 94°C for 1 min, followed by 35 cycles of 15 s at 94°C, 30 s at 65°C, and 15 min at 68°C. Final elongation entailed 10 min at 72°C. The LR-PCR was followed by nested PCR using a subset of primers described in **Table 2** (Thermo Fisher Scientific Inc., Waltham, MA). The amplification primers were used in a final volume of 20 μL, containing 0.5 μM each primer, 1× AmpliTaq Gold PCR Master Mix (Thermo Fisher Scientific Inc., Waltham, MA). Cycling conditions were as follows: initial denaturation of 95°C for 15 min, followed by 30 cycles of 30 s at 95°C, 30 s at 60°C, and 45 s at 72°C. Final elongation entailed 9 min at 72°C. Amplification was evaluated on 2% agarose gel stained with GelRed (Biotium, Hayward, CA).

The amplified samples were purified using the ExoSap enzyme protocol (Thermo Fisher Scientific Inc., Waltham, MA) to perform Sanger sequencing procedures in the ABI 3130 Genetic Analyzer Applied Biosystem platform (Life Technologies). After sequencing, specific genomic coordinates were evaluated in the electropherogram using the software CLC (QIAGEN, Inc.), which allowed us to discriminate between the presence of variants detected in the *PMS2* gene or its possible presence in the pseudogene (*PMS2CL*), demonstrating that LR-PCR can be used to amplify the *PMS2* gene and avoid interference of its pseudogene counterparts through the use of anchoring primers exclusive to the *PMS2* gene.

### Molecular analysis

#### Variant classification

All variants were annotated according to HGVS (Sequence Variant Nomenclature) recommendations. The variants were interpreted considering the clinical features of patients and the American College of Medical Genetics (ACMG) and Association for Molecular Pathology (AMP) variant classification protocol (15). Databases such as ClinVar (https://www.ncbi.nlm.nih.gov/clinvar/), ClinGen (https://clinicalgenome.org), HGMD (Human Gene Mutation Database https://www.hgmd.cf.ac.uk/ac/index.php) , Varsome (https://varsome.com/), gnomAD (https://gnomad.broadinstitute.org/), dbSNP (https://www.ncbi.nlm.nih.gov/snp/), and Abraom—variant database from Brazilian population (http://abraom.ib.usp.br/) were consulted for clinical variant interpretation assessment.

## Results

**Figure 1:**
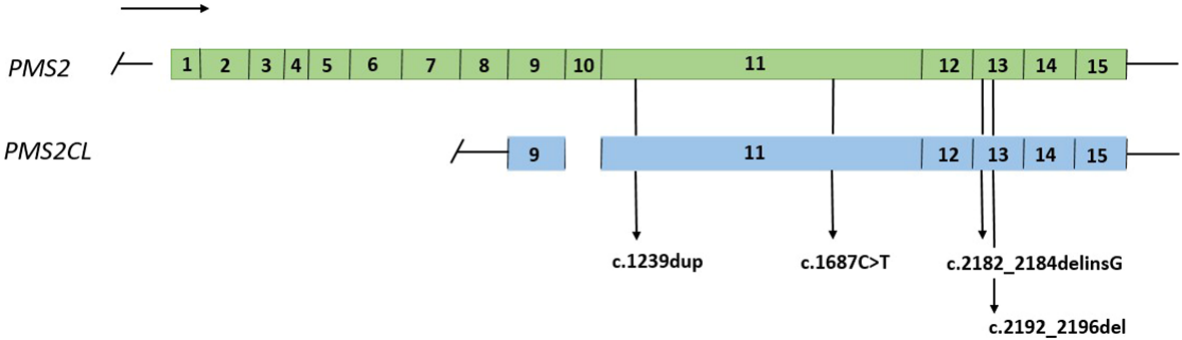
Representation of the *PMS2* gene and the *PMS2CL* pseudogene, regions of high homology and variants detected in exons 11 and 13.

A total of 74 samples with a *PMS2* GPV detected by NGS in exons with high homology with the *PMS2CL* pseudogene were evaluated. Four different GPVs were identified in exons 11 and 13 (**Figure 1**). Of these, the most common variant detected in 68 samples was NM_000535.6: c.2182_2184delinsG (p.Thr728Alafs*7) (ClinVar ID: 231999, VCV000231999.10), located in exon 13 of the *PMS2* gene, according to the NGS mapping pipeline. This variant causes a translational frameshift with a predicted stop codon and has been reported in the literature to be associated with LS (16). All detected GPVs are described in **Table 3**.

**Table 3:** Details of variants identified in the *PMS2* gene in the region of high homology with the *PMS2CL* pseudogene.

ID	Gene	Transcript	Nucleotide	Protein	VAF (%)	Exon	dbSNP	Long-range PCR result
LR001	PMS2	NM_000535.6	c.2182_2184delinsG	p.Thr728Alafs*7	29.78	13	rs1554294508	Negative
LR002	PMS2	NM_000535.6	c.2182_2184delinsG	p.Thr728Alafs*7	20.03	13	rs1554294508	Negative
LR003	PMS2	NM_000535.6	c.2182_2184delinsG	p.Thr728Alafs*7	35.25	13	rs1554294508	Negative
LR004	PMS2	NM_000535.6	c.2182_2184delinsG	p.Thr728Alafs*7	31.38	13	rs1554294508	Negative
LR005	PMS2	NM_000535.6	c.2182_2184delinsG	p.Thr728Alafs*7	21.74	13	rs1554294508	Negative
LR006	PMS2	NM_000535.6	c.2182_2184delinsG	p.Thr728Alafs*7	16.08	13	rs1554294508	Negative
LR007	PMS2	NM_000535.6	c.2182_2184delinsG	p.Thr728Alafs*7	34.59	13	rs1554294508	Negative
LR008	PMS2	NM_000535.6	c.2182_2184delinsG	p.Thr728Alafs*7	38.20	13	rs1554294508	Negative
LR009	PMS2	NM_000535.6	c.2182_2184delinsG	p.Thr728Alafs*7	32.75	13	rs1554294508	Negative
LR010	PMS2	NM_000535.6	c.2182_2184delinsG	p.Thr728Alafs*7	38.96	13	rs1554294508	Negative
LR011	PMS2	NM_000535.6	c.2182_2184delinsG	p.Thr728Alafs*7	35.36	13	rs1554294508	Negative
LR012	PMS2	NM_000535.6	c.2182_2184delinsG	p.Thr728Alafs*7	15.67	13	rs1554294508	Negative
LR013	PMS2	NM_000535.6	c.2182_2184delinsG	p.Thr728Alafs*7	15.80	13	rs1554294508	Negative
LR014	PMS2	NM_000535.6	c.2182_2184delinsG	p.Thr728Alafs*7	20.20	13	rs1554294508	Negative
LR015	PMS2	NM_000535.6	c.2182_2184delinsG	p.Thr728Alafs*7	21.11	13	rs1554294508	Negative
LR016	PMS2	NM_000535.6	c.2182_2184delinsG	p.Thr728Alafs*7	22.39	13	rs1554294508	Negative
LR017	PMS2	NM_000535.6	c.2182_2184delinsG	p.Thr728Alafs*7	22.44	13	rs1554294508	Negative
LR018	PMS2	NM_000535.6	c.2182_2184delinsG	p.Thr728Alafs*7	32.15	13	rs1554294508	Negative
LR019	PMS2	NM_000535.6	c.2182_2184delinsG	p.Thr728Alafs*7	32.23	13	rs1554294508	Negative
LR020	PMS2	NM_000535.6	c.2182_2184delinsG	p.Thr728Alafs*7	21.04	13	rs1554294508	Negative
LR021	PMS2	NM_000535.6	c.2182_2184delinsG	p.Thr728Alafs*7	18.17	13	rs1554294508	Negative
LR022	PMS2	NM_000535.6	c.2182_2184delinsG	p.Thr728Alafs*7	33.96	13	rs1554294508	Negative
LR023	PMS2	NM_000535.6	c.2182_2184delinsG	p.Thr728Alafs*7	36.93	13	rs1554294508	Negative
LR024	PMS2	NM_000535.6	c.2182_2184delinsG	p.Thr728Alafs*7	22.02	13	rs1554294508	Negative
LR025	PMS2	NM_000535.6	c.2182_2184delinsG	p.Thr728Alafs*7	31.89	13	rs1554294508	Negative
LR026	PMS2	NM_000535.6	c.2182_2184delinsG	p.Thr728Alafs*7	17.09	13	rs1554294508	Negative
LR027	PMS2	NM_000535.6	c.2182_2184delinsG	p.Thr728Alafs*7	24.16	13	rs1554294508	Negative
LR028	PMS2	NM_000535.6	c.2182_2184delinsG	p.Thr728Alafs*7	28.20	13	rs1554294508	Negative
LR029	PMS2	NM_000535.6	c.2182_2184delinsG	p.Thr728Alafs*7	38.47	13	rs1554294508	Negative
LR030	PMS2	NM_000535.6	c.2182_2184delinsG	p.Thr728Alafs*7	14.20	13	rs1554294508	Negative
LR031	PMS2	NM_000535.6	c.2182_2184delinsG	p.Thr728Alafs*7	18.50	13	rs1554294508	Negative
LR032	PMS2	NM_000535.6	c.2182_2184delinsG	p.Thr728Alafs*7	17.60	13	rs1554294508	Negative
LR033	PMS2	NM_000535.6	c.2182_2184delinsG	p.Thr728Alafs*7	18.60	13	rs1554294508	Negative
LR034	PMS2	NM_000535.6	c.2182_2184delinsG	p.Thr728Alafs*7	21.00	13	rs1554294508	Negative
LR035	PMS2	NM_000535.6	c.2182_2184delinsG	p.Thr728Alafs*7	19.30	13	rs1554294508	Negative
LR036	PMS2	NM_000535.6	c.2182_2184delinsG	p.Thr728Alafs*7	18.70	13	rs1554294508	Negative
LR037	PMS2	NM_000535.6	c.2182_2184delinsG	p.Thr728Alafs*7	19.10	13	rs1554294508	Negative
LR038	PMS2	NM_000535.6	c.2182_2184delinsG	p.Thr728Alafs*7	18.80	13	rs1554294508	Negative
LR039	PMS2	NM_000535.6	c.2182_2184delinsG	p.Thr728Alafs*7	10.70	13	rs1554294508	Negative
LR040	PMS2	NM_000535.6	c.2182_2184delinsG	p.Thr728Alafs*7	23.20	13	rs1554294508	Negative
LR041	PMS2	NM_000535.6	c.2182_2184delinsG	p.Thr728Alafs*7	14.50	13	rs1554294508	Negative
LR042	PMS2	NM_000535.6	c.2182_2184delinsG	p.Thr728Alafs*7	20.30	13	rs1554294508	Negative
LR043	PMS2	NM_000535.6	c.2182_2184delinsG	p.Thr728Alafs*7	22.70	13	rs1554294508	Negative
LR044	PMS2	NM_000535.6	c.2182_2184delinsG	p.Thr728Alafs*7	39.70	13	rs1554294508	Negative
LR045	PMS2	NM_000535.6	c.2182_2184delinsG	p.Thr728Alafs*7	35.50	13	rs1554294508	Negative
LR046	PMS2	NM_000535.6	c.2182_2184delinsG	p.Thr728Alafs*7	15.90	13	rs1554294508	Negative
LR047	PMS2	NM_000535.6	c.2182_2184delinsG	p.Thr728Alafs*7	21.60	13	rs1554294508	Negative
LR048	PMS2	NM_000535.6	c.2182_2184delinsG	p.Thr728Alafs*7	21.00	13	rs1554294508	Negative
LR049	PMS2	NM_000535.6	c.2182_2184delinsG	p.Thr728Alafs*7	23.50	13	rs1554294508	Negative
LR050	PMS2	NM_000535.6	c.2182_2184delinsG	p.Thr728Alafs*7	34.40	13	rs1554294508	Negative
LR051	PMS2	NM_000535.6	c.2182_2184delinsG	p.Thr728Alafs*7	23.00	13	rs1554294508	Negative
LR052	PMS2	NM_000535.6	c.2182_2184delinsG	p.Thr728Alafs*7	21.90	13	rs1554294508	Negative
LR053	PMS2	NM_000535.6	c.2182_2184delinsG	p.Thr728Alafs*7	20.40	13	rs1554294508	Negative
LR054	PMS2	NM_000535.6	c.2182_2184delinsG	p.Thr728Alafs*7	18.10	13	rs1554294508	Negative
LR055	PMS2	NM_000535.6	c.2182_2184delinsG	p.Thr728Alafs*7	36.10	13	rs1554294508	Negative
LR056	PMS2	NM_000535.6	c.2182_2184delinsG	p.Thr728Alafs*7	22.50	13	rs1554294508	Negative
LR057	PMS2	NM_000535.6	c.2182_2184delinsG	p.Thr728Alafs*7	22.40	13	rs1554294508	Negative
LR058	PMS2	NM_000535.6	c.2182_2184delinsG	p.Thr728Alafs*7	36.40	13	rs1554294508	Negative
LR059	PMS2	NM_000535.6	c.2182_2184delinsG	p.Thr728Alafs*7	21.60	13	rs1554294508	Negative
LR060	PMS2	NM_000535.6	c.2182_2184delinsG	p.Thr728Alafs*7	19.70	13	rs1554294508	Negative
LR061	PMS2	NM_000535.6	c.2182_2184delinsG	p.Thr728Alafs*7	25.50	13	rs1554294508	Negative
LR062	PMS2	NM_000535.6	c.2182_2184delinsG	p.Thr728Alafs*7	20.10	13	rs1554294508	Negative
LR063	PMS2	NM_000535.6	c.2182_2184delinsG	p.Thr728Alafs*7	34.20	13	rs1554294508	Negative
LR064	PMS2	NM_000535.6	c.2182_2184delinsG	p.Thr728Alafs*7	28.70	13	rs1554294508	Negative
LR065	PMS2	NM_000535.6	c.2182_2184delinsG	p.Thr728Alafs*7	25.30	13	rs1554294508	Negative
LR066	PMS2	NM_000535.6	c.2182_2184delinsG	p.Thr728Alafs*7	21.00	13	rs1554294508	Negative
LR067	PMS2	NM_000535.6	c.2182_2184delinsG	p.Thr728Alafs*7	19.80	13	rs1554294508	Negative
LR068	PMS2	NM_000535.6	c.2182_2184delinsG	p.Thr728Alafs*7	26.60	13	rs1554294508	Negative
LR069	PMS2	NM_000535.6	c.1687C>T	p.Arg563*	46.90	11	rs587778618	Positive
LR070	PMS2	NM_000535.7	c.1239dup	p.Asp414Argfs*44	51.55	11	rs267608159	Positive
LR071	PMS2	NM_000535.7	c.1239dup	p.Asp414Argfs*44	53.91	11	rs267608159	Positive
LR072	PMS2	NM_000535.7	c.1239dup	p.Asp414Argfs*44	48.10	11	rs267608159	Positive
LR073	PMS2	NM_000535.7	c.2192_2196del	p.Leu731Cysfs*3	21.00	13	rs63750695	Negative
LR074	PMS2	NM_000535.7	c.2192_2196del	p.Leu731Cysfs*3	31.00	13	rs63750695	Positive

It was not possible to correlate the molecular findings with the tumor MMR status (immunohistochemical) or tissue microsatellite instability analysis.

We did not have access to the correlated data or clinical information of the patients evaluated in this study. All the 68 patients harboring variant c.2182_2184delinsG, detected through NGS, have failed to confirm it by LR-PCR, indicating the absence of this variant in *PMS2*. The mean variant allele frequency (VAF) of these variants was 24.7% (ranging from 10.7 to 39.7%), and the median was 22.2% (**Table 3**).

Of the other six GPV detected, four are located in exon 11 and two in exon 13 (**Table 3**). Five samples evaluated by LR-PCR were confirmed and considered positive result. Three patients had the following confirmed variant NM_000535.7:c.1239dup (ClinVar ID: 216072, VCV000216072.32), located in exon 11 of *PMS2*. The mean VAF of these variants was 51.18% (ranging from 48.1 to 53.91%), and the median was 51.55%. The variant NM_000535.6:c.1687C>T (ClinVar ID: 135067, VCV000135067.29) was detected in one patient with VAF of 46.9% and was confirmed by LR-PCR.

The variant NM_000535.7:c.2192_2196del (ClinVar ID: 91331, VCV000091331.38) was detected in two patients and confirmed in one case. The mean VAF was 26%.

In summary, of the 74 GPV identified, five (6.8%) were confirmed by LR-PCR. Conversely, the other 69 patients (93.2%) who did not confirm the presence of the variant after LR-PCR had the diagnosis of LS ruled out by molecular mechanisms associated with the *PMS2* gene, not excluding the possibility of other clinical criteria involved with this diagnosis.

Considering the five variants confirmed by LR-PCR, we found a mean VAF of 46.3% versus 24.6% of the other 69 unconfirmed variants.

## Discussion and conclusion

NGS has some limitations, and the analysis of genes with high identity to pseudogenes is one of them. This is an important issue because the presence of the pseudogene can result in false positive or negative tests, thereby affecting clinical practice and genetic counseling. Thus, the use of different approaches is necessary to avoid interference with data interpretation, which could lead to misleading conduct.

The analysis of *PMS2* variants by NGS is very complex. Even considering that the NGS approach can detect variants along all the coding segments of this gene, when a pathogenic variant is detected, the correct clinical interpretation is very challenging because of the high homology of *PMS2* to its counterparts, such as the non-expressed *PMS2CL* pseudogene (17, 18).

The evaluation of *PMS2* gene is neglected by many laboratories, due to the methodological difficulties in reporting reliable variants and not ensuring that the variant has been detected in the gene. Many commercial laboratories do not analyze the regions of these *PMS2* pseudogenes, consequently generating incomplete analysis of this gene. However, some laboratories report GPV in these regions of high homology, but without confirmation using other techniques.

Although the *PMS2* gene has low penetrance, a reliable identification of the presence of pathogenic variants in this gene is fundamental for the correct management of LS and genetic counseling.

In our experience, more than 90% of the pathogenic variants identified by NGS in the *PMS2* gene in exons with high identity to pseudogenes were not confirmed using LR-PCR. We observed that variants confirmed by LR-PCR presented a higher VAF, near 50%. In contrast, unconfirmed variants had lower VAF, indicating that variants in the actual gene have higher VAF.

It is important to note that c.2182_2184delinsG variant identified in our cohort was not confirmed to be in the *PMS2* gene in any of the patients. This variant is reported in ClinVar database as conflicting, was previously described as a deleterious mutation in a study of African-American patients with LS, and has been widely reported by laboratories as a pathogenic variant associated with the LS phenotype (16). According to Chong et al. (2020), this variant was incorrectly assigned to *PMS2* in a sample of patients, suggesting reclassification and caution when interpreting these variants (19). Although we studied variants only in exons 11 and 13, this methodology was developed to confirm variants in other regions with homology to *PMS2* gene.

Our findings strongly support this suggestion, and we recommend a pathogenic variant classification only if the variant is at the *PMS2* gene evaluated by a LR-PCR. Thus, there are benefits for patients because the diagnosis of LS is excluded, avoiding unnecessary screening and even unequivocal indication of hysterectomy and risk-reducing salpingo-oophorectomy (20).

In conclusion, the use of LR-PCR was demonstrated to be a reliable approach for accurate molecular analysis of *PMS2* gene variants in segments with high homology with the *PMS2CL* pseudogene. We highlight that our laboratory is a pioneer in the diagnostic complementation of the *PMS2* gene in Brazil, directly contributing to more assertive molecular diagnosis. Our results indicate that using confirmation strategies such as LR-PCR in those segments is essential to avoid misdiagnosis of LS, directly impacting the genetic counseling of these patients and their families, since the correct molecular diagnosis can avoid inappropriate clinical management.

## Data availability statement

The datasets presented in this study can be found in online repositories. The names of the repository/repositories and accession number(s) can be found in the article/supplementary material.

## Ethics statement

The studies involving humans were approved by Human Research Ethics Committee (protocol number NP_614) - Fleury S.A. The studies were conducted in accordance with the local legislation and institutional requirements. The participants provided their written informed consent to participate in this study.

## Author contributions

DP: Conceptualization, Data curation, Formal analysis, Investigation, Methodology, Project administration, Software, Supervision, Validation, Visualization, Writing – original draft, Writing – review & editing. TL: Conceptualization, Data curation, Formal analysis, Investigation, Methodology, Validation, Writing – original draft, Writing – review & editing. RS: Methodology, Validation, Writing – review & editing. JC: Writing – review & editing. CP-A: Writing – review & editing. IS-F: Writing – review & editing. PS: Writing – review & editing. MM-N: Methodology, Validation, Writing – review & editing. CM: Project administration, Supervision, Writing – review & editing. WB: Project administration, Supervision, Writing – review & editing.
